# Recent Advances in Natural Products for Colitis: Mechanisms and Translational Perspectives

**DOI:** 10.3390/vetsci13060532

**Published:** 2026-05-29

**Authors:** Fulin Jin, Yaning Shi, Lijun Wang, Tinghong Kuang, Keyan Ren, Jiaye Xu, Yifan Zhang, Changchang Niu, Ji Cheng, Shifeng Pan

**Affiliations:** 1 College of Veterinary Medicine, Yangzhou University, Yangzhou 225009, China; 13851118926@163.com (F.J.); 13401420552@163.com (J.X.);; 2 Jiangsu Co-Innovation Center for Prevention and Control of Important Animal Infectious Diseases and Zoonoses, Yangzhou University, Yangzhou 225009, China

**Keywords:** colitis, natural products, intestinal barrier, oxidative stress, gut microbiota, inflammation, translational prospects

## Abstract

Colitis is a chronic relapsing inflammatory disorder characterized by persistent mucosal inflammation, epithelial barrier disruption, oxidative stress, immune dysregulation, and gut microbiota imbalance. Although current therapies have improved disease management, their long-term use is still limited by incomplete response, relapse, adverse effects, and high cost. Natural products have attracted increasing attention as promising candidates for colitis intervention because of their structural diversity, broad bioactivity, and multitarget regulatory potential. In veterinary medicine, colitis and colitis-related intestinal inflammatory disorders are also clinically relevant in companion animals, horses, and food-producing animals, where they contribute to diarrhea, impaired intestinal barrier function, reduced performance, treatment costs, and antimicrobial-use concerns. Therefore, natural products may have additional value as adjunctive, nutritional, or microbiota-modulating strategies in veterinary and comparative gastroenterology. This review summarizes the major categories of natural products investigated for colitis, including flavonoids, polyphenols, alkaloids, terpenoids, saponins, polysaccharides, plant extracts, and traditional herbal formulas, and discusses their principal mechanisms of action, such as anti-inflammatory activity, attenuation of oxidative stress, restoration of intestinal barrier integrity, modulation of immune responses, regulation of gut microbiota, and control of programmed cell death. We also highlight the major limitations of current research, including overreliance on preclinical models, insufficient target validation, poor bioavailability, and limited clinical evidence. Overall, natural products represent an important source of therapeutic candidates for colitis, but their clinical translation will require deeper mechanistic studies, standardized evaluation, and well-designed clinical investigations. Importantly, future studies should distinguish phenotype-associated changes from causal mechanisms by integrating direct target engagement, functional rescue experiments, microbiota-causality validation, and standardized pharmacokinetic and safety assessment.

## Simple Summary

Intestinal inflammation is a common health problem in both people and animals. In animals, it can cause diarrhea, poor growth, reduced productivity, higher treatment costs, and welfare problems. In food-producing animals, repeated intestinal disease may also increase the need for medication, including antibiotics, which is a concern for both animal production and public health. Current treatments can be helpful, but they do not work well for all cases and may be limited by relapse, side effects, or cost. For these reasons, there is growing interest in natural products, such as plant-derived compounds, herbal extracts, and natural sugars from plants, fungi, or microbes, as supportive approaches for maintaining intestinal health. This review summarizes studies showing that these natural products may help calm intestinal inflammation, protect the gut lining, support a healthier gut microbial balance, and improve the body’s response to intestinal injury. These findings suggest that natural products may have value not only for human intestinal diseases but also for veterinary medicine, especially as nutritional or complementary strategies to support gut health in companion animals, horses, and farm animals. However, most current evidence still comes from laboratory and animal experiments, and many products have not yet been carefully tested for safety, absorption, effective dose, or real clinical benefit. Therefore, stronger research and well-designed clinical studies are needed before these products can be widely recommended. This review may help researchers, veterinarians, and clinicians better evaluate which natural products are promising and how they could be developed responsibly for intestinal health management.

## 1. Introduction

Colitis is a chronic and relapsing inflammatory disorder of the colon, clinically represented most prominently by ulcerative colitis (UC) and commonly modeled in experimental studies by chemically induced colitis models such as dextran sulfate sodium (DSS)- or TNBS-induced colitis. Its onset and progression are closely associated with genetic susceptibility, environmental exposure, host immune dysregulation, epithelial barrier disruption, oxidative stress, and gut microbiota imbalance [1,2,3]. Clinically, patients with active colitis often present with abdominal pain, diarrhea, mucous and bloody stools, and weight loss, whereas persistent or severe inflammation may lead to mucosal ulceration, intestinal fibrosis, stricture formation, and an increased risk of inflammation-associated tumorigenesis [4,5]. Because of its chronic relapsing course and substantial impact on long-term disease management, colitis remains a major challenge in gastrointestinal medicine. Beyond human ulcerative colitis, colitis and colitis-related intestinal inflammatory disorders are highly relevant to veterinary medicine. In dogs and cats, chronic enteropathies and inflammatory bowel disease-like conditions are associated with persistent gastrointestinal signs, mucosal immune activation, altered gut microbiota, and variable responses to diet, antimicrobials, probiotics, and immunomodulatory therapy [6,7,8,9]. In horses, acute colitis and diarrhea are clinically important because they may progress rapidly, are associated with systemic inflammation, fluid loss, dysbiosis, and substantial morbidity or mortality [10,11]. In food-producing animals, especially weaned pigs and calves, intestinal inflammation and diarrhea impair epithelial barrier development, nutrient absorption, growth performance, welfare, and production efficiency [12]. Therefore, although the terminology and clinical context differ among species, intestinal inflammation in animals shares several mechanistic features with human colitis, including barrier disruption, microbiota dysbiosis, oxidative stress, and immune dysregulation.

Although substantial progress has been made in treatment, IBD remains incurable. Current therapeutic options, including 5-aminosalicylic acid agents, corticosteroids, immunosuppressants, biologics, and small-molecule targeted therapies, are effective for inducing and maintaining remission in many patients [4,13]. However, clinical practice still faces major challenges such as primary or secondary nonresponse, relapse after drug withdrawal, cumulative adverse effects, and high treatment costs. More importantly, colitis is not caused by the dysregulation of a single inflammatory pathway, but rather results from the interplay of inflammatory cascades, mucosal barrier disruption, oxidative stress imbalance, gut microbiota dysbiosis, and host metabolic abnormalities [4,14,15,16]. This complexity also means that single-target interventions are often insufficient to fully meet the demands of long-term disease management.

Against this background, natural products have gradually become a major focus in colitis prevention and treatment research because of their wide availability, structural diversity, broad bioactivity, and multitarget regulatory potential. In recent years, a large number of preclinical studies have shown that naturally derived bioactive compounds, including flavonoids, polyphenols, alkaloids, terpenoids, saponins, polysaccharides, plant extracts, and traditional herbal formulas, can improve colitis phenotypes to varying degrees while simultaneously acting on multiple key pathological processes, such as suppressing inflammatory signaling, alleviating oxidative stress, repairing the intestinal mucosal barrier, regulating the immune microenvironment, and remodeling the gut microbiota and metabolic networks [2,13].

Based on this background, this review provides a comprehensive overview of recent advances in natural products for colitis. First, starting from the major pathophysiological basis underlying the development of colitis, we outline the theoretical basis for the intervention of natural products and classify the major research subjects, including flavonoids, polyphenols, alkaloids, terpenoids and saponins, polysaccharides, plant extracts, and traditional herbal formulas. Second, we focus on summarizing the mechanisms by which natural products exert their protective effects, including suppression of inflammatory signaling, attenuation of oxidative stress, restoration of the intestinal mucosal barrier, regulation of the immune microenvironment, remodeling of the gut microbiota and its metabolic networks, and modulation of programmed cell death. Finally, in light of the current research landscape, we analyze the major limitations in this field and discuss the translational prospects of natural products in combination therapy, delivery system optimization, and personalized application.

## 2. Pathophysiological Basis and Major Categories of Natural Products for the Treatment of Colitis

### 2.1. Pathophysiological Basis for the Intervention of Natural Products in Colitis

**Figure 1:**
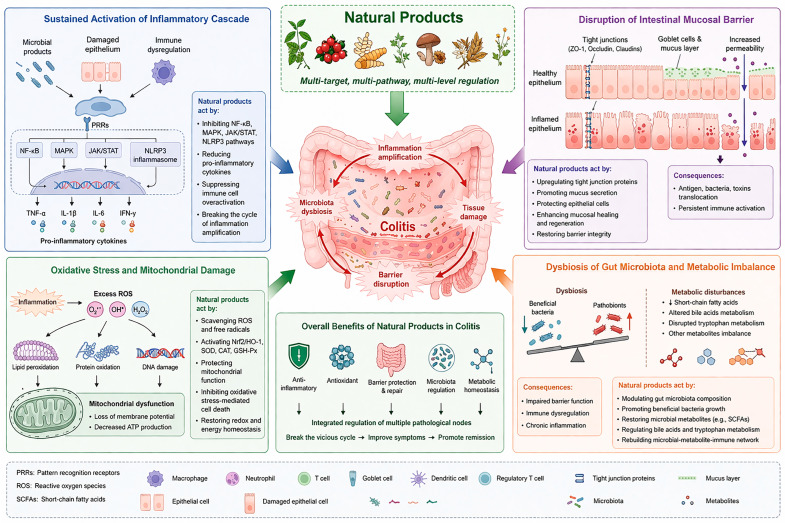
The mechanism of natural products in treating colitis.

Colitis is not a localized lesion caused by the abnormal expression of a single inflammatory factor, but rather a complex disease process shaped by the interaction of multiple upstream risk factors and downstream pathological events. In addition to genetic susceptibility, age, dietary pattern, environmental exposure, infection, medication use, and psychological stress may all influence disease onset, severity, and progression. At the mechanistic level, host immune dysregulation, intestinal mucosal barrier disruption, enhanced oxidative stress, gut microbiota imbalance, and metabolic reprogramming further amplify inflammation and tissue injury. Because colitis is driven by both multifactorial risk determinants and multi-level pathological abnormalities, single-target therapies often act only at a limited stage of disease progression and are insufficient to simultaneously achieve inflammation control, tissue repair, and microecological reconstruction. In this context, natural products have attracted sustained attention because their multitarget, multipathway, and multi-level regulatory properties make them theoretically more suitable for the integrated management of such complex chronic inflammatory diseases (Figure 1).

#### 2.1.1. Persistent Inflammatory Signaling in Colitis

Persistent activation of inflammatory signaling is one of the most fundamental pathological bases of colitis. The earliest and most common mechanistic entry point in studies of natural products for colitis has therefore been the inhibition of inflammatory signaling pathways and the reduction in pro-inflammatory mediator release. A large number of preclinical studies have shown that flavonoids, polyphenols, alkaloids, and various plant extracts can reduce, to different extents, the expression of inflammation-related molecules such as TNF-α, IL-1β, IL-6, COX-2, and iNOS, and these effects are often associated with the regulation of pathways including NF-κB [17], MAPK [18], JAK/STAT, and the NLRP3 [19] inflammasome.

##### NF-κB Signaling Pathway

NF-κB is a central transcriptional regulatory axis that links external inflammatory stimuli to the transcription of downstream inflammatory genes and plays a pivotal role in colitis [20,21]. Following epithelial injury, stimulation by microbe-associated molecular patterns, and activation of local immune cells, IκBα is phosphorylated and degraded, allowing the p65/p50 subunits of the NF-κB complex to translocate into the nucleus and initiate the expression of multiple pro-inflammatory genes, including TNF-α, IL-1β, and IL-6 [22,23]. The intervention of natural products in this pathway is mainly reflected by inhibition of IKK/IκBα activation, reduction in p65 phosphorylation and nuclear translocation, and consequent attenuation of inflammatory amplification [24,25]. NF-κB is frequently examined in colitis research not only because it is consistently activated and experimentally accessible, but also because many of the reported anti-inflammatory effects of natural products are at least partly associated with the modulation of this classical signaling pathway [25,26,27,28].

##### NLRP3 Inflammasome-Related Pathway

The NLRP3 inflammasome has emerged as one of the fastest-growing mechanistic hotspots in the study of natural products for colitis [29,30]. The NLRP3 inflammasome more directly connects inflammatory responses with inflammatory cell death, especially through the maturation and release of IL-1β and IL-18 and through processes related to pyroptosis. Many natural products have been reported to alleviate local colonic inflammation by suppressing NLRP3 expression, blocking inflammasome assembly, or reducing Caspase-1 activity [31,32,33,34]. Because this mechanism combines features of both inflammatory regulation and cell-death regulation, it is particularly attractive when explaining the multi-layered intervention effects of natural products.

#### 2.1.2. Oxidative Stress and Endogenous Antioxidant Defense

Oxidative stress is a major pathological layer in colitis and is mainly characterized by excessive production of reactive oxygen species, lipid peroxidation, and insufficient endogenous antioxidant defense. Under inflammatory conditions, excessive ROS can damage lipids, proteins, and DNA, thereby aggravating epithelial injury and mucosal inflammation [35,36,37,38]. Natural products may reduce oxidative injury by decreasing ROS accumulation, limiting lipid peroxidation, and activating endogenous antioxidant pathways such as the Nrf2/HO-1 axis [39,40].

##### Reduction in ROS Levels and Lipid Peroxidation Damage

Reduction in ROS levels is one of the most common oxidative stress-related effects of natural products in colitis. A large number of studies have shown that various flavonoids, polyphenols, and plant extracts can markedly lower oxidative injury indices such as ROS and MDA in colonic tissues or cells, while increasing total antioxidant capacity. Because these results are usually accompanied by reduced inflammatory mediator levels and improved tissue damage, they are often regarded as direct evidence for the “antioxidant protection” of natural products [35,36]. However, it should be emphasized that a simple reduction in ROS or MDA does not automatically mean that a natural product directly acts on oxidative stress pathways. Therefore, decreases in ROS, MDA, or lipid peroxidation markers should be interpreted as evidence of reduced oxidative injury rather than direct proof that a compound specifically targets oxidative stress pathways.

##### Activation of Endogenous Antioxidant Systems Such as Nrf2/HO-1

Compared with direct ROS scavenging, activation of endogenous antioxidant systems such as Nrf2/HO-1 suggests that natural products may more deeply participate in the regulation of cellular antioxidant defense networks. Nrf2 is a central transcription factor in oxidative stress responses, and once activated, it induces the expression of multiple antioxidant and cytoprotective genes, including HO-1 and NQO1. Many natural products have been reported to promote Nrf2 nuclear translocation and enhance HO-1 expression, thereby increasing endogenous antioxidant capacity and alleviating inflammatory injury [39,41].

#### 2.1.3. Improvement of Mitochondrial Dysfunction and Energy Metabolic Imbalance

Mitochondrial dysfunction represents a mechanistically distinct extension of oxidative injury in colitis. Rather than simply acting as another source of ROS, damaged mitochondria contribute to epithelial dysfunction by reducing ATP production, disrupting membrane potential, impairing mitochondrial dynamics, and weakening mitochondrial quality control. Therefore, natural products that preserve mitochondrial function may support mucosal repair not only by reducing oxidative damage, but also by maintaining cellular energy homeostasis and epithelial survival [42,43,44,45].

The importance of this direction lies in the fact that it pushes research on natural products in colitis beyond “improvement of inflammatory indices” toward “maintenance of cellular energy homeostasis”. Nevertheless, most current studies still remain at the level of observing mitochondrial phenotypes, such as changes in membrane potential, ATP, or ROS. Work that truly extends into mitochondrial quality control, mitochondria–endoplasmic reticulum interactions, or immunometabolic reprogramming is still limited. Thus, although mitochondrial protective mechanisms have become a new extension point in natural product research, their systematic depth remains clearly insufficient.

##### Repair of the Intestinal Mucosal Barrier and Promotion of Mucosal Healing

Compared with simple inflammation suppression, repair of the intestinal mucosal barrier is more closely aligned with long-term control of colitis and prevention of relapse. In colitis, barrier disruption is not only a consequence of inflammation, but also a key driver of persistent inflammation [46,47]. Natural products can improve colitis not only because they suppress inflammatory pathways, but also because many compounds can directly or indirectly promote barrier recovery, including restoration of tight junction proteins, protection of goblet cells and the mucus layer, stimulation of epithelial regeneration, and reduction in intestinal permeability. Existing reviews generally regard “barrier repair” as one of the major advantages of natural products over purely immunosuppressive drugs [48,49,50,51].

##### Restoration of Tight Junction Protein Expression

Under colitic conditions, tight junction integrity is disrupted, as reflected by reduced expression of proteins such as ZO-1, Occludin, and Claudins, together with goblet cell loss, insufficient mucus secretion, and increased intestinal permeability. These alterations facilitate the continuous entry of microbial products and inflammatory stimuli into the mucosa and submucosa, thereby further promoting local immune activation and sustaining mucosal inflammation [52,53]. In this context, the ability of natural products to simultaneously improve barrier function has become an important dimension in evaluating their preventive and therapeutic potential.

##### Protection of Goblet Cells and the Mucus Barrier

Goblet cells and the mucus layer are important components of the first line of defense in the intestine. Under colitic conditions, reduction in goblet cells and depletion of mucus components such as MUC2 expose the epithelium directly to microbiota and inflammatory stimuli, thereby aggravating tissue damage. Some natural products have been shown to increase goblet cell numbers, promote mucus secretion, or restore MUC2 expression, which is highly relevant for reducing direct microbial contact with the epithelium [54]. However, studies in this area are usually limited to histological staining or MUC2 detection, and more refined quantification of mucus secretion kinetics, mucus layer thickness, and functional protection remains lacking. Thus, goblet cell and mucus barrier protection is an attractive mechanistic point in natural product research for colitis, but the precision of evidence still has room for improvement.

#### 2.1.4. Regulation of Immune Cell Function and Remodeling of the Inflammatory Microenvironment

Immune-cell dysfunction represents a cellular level of inflammatory amplification in colitis. Unlike Section 2.1.1, which focuses on intracellular inflammatory signaling pathways, this section emphasizes how natural products regulate immune-cell composition and function within the mucosal microenvironment. In particular, macrophage activation, Treg/Th17 imbalance, neutrophil infiltration, and excessive innate immune activation contribute to persistent mucosal injury. Natural products may therefore alleviate colitis not only by suppressing inflammatory mediators, but also by reshaping the immune-cell landscape toward a state more favorable for inflammation resolution and mucosal repair.

##### Regulation of Macrophage Activation and Polarization

Macrophages are key regulators of the local inflammatory microenvironment in colitis. M1 macrophages tend to release pro-inflammatory mediators and amplify tissue injury, whereas M2 macrophages are more associated with anti-inflammatory activity and tissue repair. Many natural products have been reported to suppress M1-associated molecules and promote M2 polarization, thereby achieving parallel effects on inflammation suppression and tissue repair [55,56,57,58].

##### Regulation of Immune Balance

Treg/Th17 imbalance is regarded as an important feature of chronic immune dysregulation in colitis. Treg cells help suppress excessive inflammation and maintain tolerance, whereas Th17 cells are often associated with inflammatory amplification and mucosal injury. Various natural products have been reported to increase Treg levels, decrease Th17 responses, or rebalance the two, thereby alleviating inflammation and restoring immune homeostasis [59,60]. However, a common problem in this area is that many studies infer modulation of Treg/Th17 balance merely from changes in IL-17, Foxp3, or related cytokines, without true functional verification of the relevant cell subsets [61]. Thus, although this mechanism is highly valuable in theory, the strength of current evidence remains quite uneven.

##### Suppression of Neutrophil Infiltration and Excessive Innate Immune Activation

Massive neutrophil infiltration is a hallmark of acute intestinal inflammation, and the reactive oxygen species, proteases, and NETs released by these cells can substantially aggravate tissue injury [62,63]. Natural products can reduce acute inflammatory damage by lowering chemokine expression, inhibiting MPO activity, or reducing neutrophil recruitment [64,65]. At the same time, they may also contribute to restoration of local immune homeostasis through regulation of dendritic cells, innate lymphoid cells, or other innate immune components.

#### 2.1.5. Remodeling of the Gut Microbiota and Regulation of Host Metabolic Networks

The gut microbiota and its metabolites play a foundational role in the initiation and progression of colitis, making the regulation of microbial and metabolic networks one of the most actively expanding directions in natural product research. Compared with classical anti-inflammatory drugs, natural products show more obvious advantages within the composite “microbiota–metabolism–host” axis, especially polysaccharides, certain polyphenols, and plant extracts, which are often considered to possess unique potential in microecological remodeling [66,67]. This axis is particularly relevant in veterinary medicine because diet, feed additives, environmental management, antibiotic exposure, and host species strongly influence intestinal microbial composition and function. In dogs, probiotic and synbiotic effects may vary according to host factors, product composition, dose, and treatment duration [68,69]. In horses, dysbiosis is closely associated with diarrhea and colitis, and microbiota-directed approaches such as fecal microbiota transplantation have been explored as potential interventions [70,71]. In weaned pigs, nutritional interventions, phytochemicals, organic acids, short-chain fatty acids, and other feed additives are widely investigated to support gut barrier function, microbiota balance, and disease resistance [72,73].

##### Regulation of Microbiota Composition and Microecological Homeostasis

A large number of studies have shown that natural products can increase beneficial bacterial abundance, suppress opportunistic pathogen expansion, and improve overall microbial structural imbalance [74,75]. Such regulation is commonly thought to reduce inflammatory stimulation, improve epithelial barrier function, and promote mucosal healing. However, a very common problem arises here: changes in microbiota composition do not mean that a microbiota-based mechanism has been proven. Many studies define microbiota remodeling as the core mechanism based solely on 16S sequencing results, without further verifying whether these changes truly drive the improved phenotype. Therefore, a more reasonable review statement is that natural products are broadly associated with optimization of microbiota composition, but whether this represents the principal mechanism requires stricter causal validation.

##### Improvement of Microbiota-Related Metabolite Profiles

In addition to microbiota composition, alterations in microbial metabolites may more directly influence host inflammatory status and epithelial function [76,77]. Short-chain fatty acids, bile acids, and tryptophan metabolites can all participate in colitis progression by regulating immunity, barrier integrity, and energy metabolism. Natural products have been reported to increase SCFAs, optimize bile acid composition, and affect tryptophan metabolic pathways, thereby producing deeper microecological regulatory effects [49,78,79,80]. From a research perspective, this area is more substantial than simple 16S analysis because it approaches the level of “functional outcomes”. From a research perspective, metabolite profiling provides functional information beyond taxonomic profiling [81,82]. Nevertheless, many reported metabolite changes still lack supplementation experiments, receptor-level blockade, or phenotype-rescue validation. Therefore, microbiota- and metabolite-related findings should be interpreted cautiously: changes in bacterial composition or metabolite abundance do not necessarily indicate that these changes drive the therapeutic phenotype [83,84]. A microbiota-dependent mechanism should ideally be supported by antibiotic depletion, fecal microbiota transplantation, germ-free or gnotobiotic models, defined-strain colonization, metabolite supplementation, or receptor-level blockade [85].

#### 2.1.6. Regulation of Programmed Cell Death and Cellular Homeostasis

Programmed cell death provides a distinct cell-fate layer through which natural products may influence colitis progression. Unlike general inflammatory signaling or oxidative injury, this section focuses on regulated cell-death processes, including apoptosis, pyroptosis, and ferroptosis, that directly affect epithelial integrity, immune activation, and tissue homeostasis [86,87]. This distinction is important because cell death-related mechanisms often overlap with inflammation and oxidative stress, but they require specific molecular evidence rather than general changes in cytokines, ROS, or histological injury.

##### Apoptosis

Apoptosis was one of the earliest forms of cell death incorporated into natural product studies of colitis. Excessive apoptosis of epithelial cells weakens barrier function and aggravates inflammation, and many natural products have therefore been reported to alleviate epithelial apoptosis by modulating the Bcl-2/Bax ratio and inhibiting Caspase-3 activation [88,89].

##### Pyroptosis

Pyroptosis is an inflammatory form of programmed cell death characterized by Caspase-1 activation, GSDMD cleavage, membrane pore formation, and release of IL-1β and IL-18. Because the NLRP3 inflammasome has already been discussed above as an inflammatory signaling platform, pyroptosis is highlighted here mainly as the downstream cell-death outcome of inflammasome activation. Natural products may attenuate epithelial and immune-cell pyroptosis by reducing GSDMD cleavage and limiting inflammasome-dependent cytokine release, thereby decreasing mucosal injury [90,91].

##### Ferroptosis

Ferroptosis is a regulated form of cell death characterized by iron-dependent lipid peroxidation and dysfunction of the GPX4/SLC7A11 antioxidant system. Although ferroptosis is closely related to oxidative stress, it should not be treated as a simple extension of ROS accumulation. In colitis studies, natural products have been reported to attenuate ferroptosis by regulating iron handling, lipid peroxidation, GPX4, SLC7A11, and related antioxidant pathways [92,93,94,95]. However, ferroptosis-specific conclusions require evidence beyond reduced ROS or MDA levels, such as lipid peroxidation-specific detection, iron accumulation analysis, GPX4/SLC7A11 validation, ferroptosis inhibitor rescue, or genetic intervention of key ferroptosis regulators.

## 3. Natural Products for Colitis Treatment: Classification, Evidence Strength, and Limitations

### 3.1. Major Categories of Natural Products for the Treatment of Colitis

**Figure 2:**
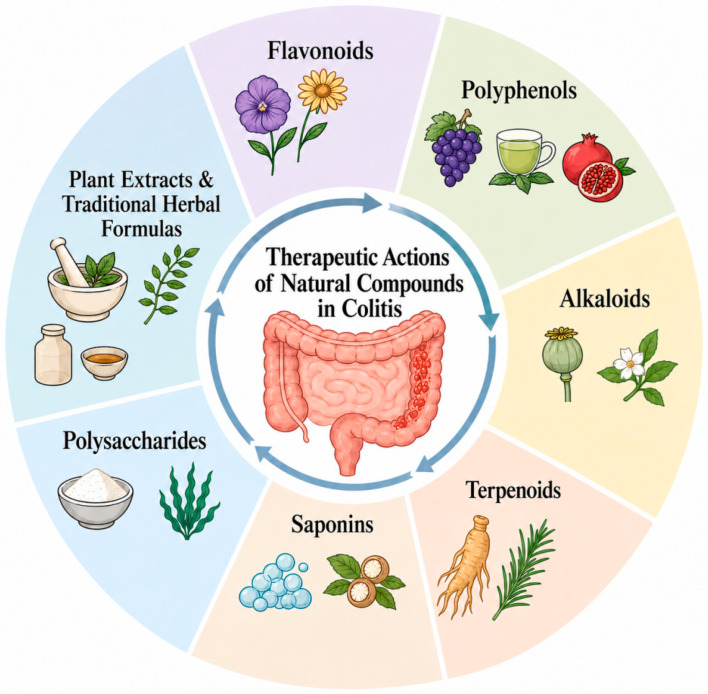
Classification of natural products for treating colitis.

From the organization of existing reviews, natural products are generally classified according to their chemical properties and sources into several major categories, including flavonoids, polyphenols, alkaloids, terpenoids and saponins, polysaccharides, as well as plant extracts and traditional herbal formulas (Figure 2). To move beyond a purely descriptive classification, Table 1 was revised to include the main experimental models, representative quantitative endpoints, mechanistic evidence, level of supporting evidence, and major limitations. The level of supporting evidence was classified as low, moderate, or relatively high according to the consistency of phenotypic improvement, the breadth of improved endpoints, the presence of dose–response evidence, and the depth of mechanistic validation.

Given the substantial heterogeneity among existing studies, a direct quantitative meta-analysis of natural products for colitis remains challenging [96]. The included studies differ markedly in colitis models, animal species and strains, sex, dose, administration route, intervention duration, disease stage, and endpoint selection [97]. Therefore, simple cross-study comparison of absolute values, such as DAI score, colon length, cytokine levels, or histological scores, may be misleading. To provide a more informative comparison, this review summarizes representative quantitative endpoints where available and further classifies the overall effect strength of different natural product categories in a semi-quantitative manner. In this framework, effect strength was evaluated based on the consistency of phenotypic improvement, the number of endpoints improved, the presence of dose–response evidence, and the depth of mechanistic validation. This approach does not replace formal meta-analysis, but it helps readers distinguish compounds with relatively robust evidence from those supported mainly by descriptive or preliminary findings (Table 1).

**Table 1:** Comparative evidence profile of representative natural products investigated for colitis.

Category	Representative Candidates	Main Experimental Models	Representative Quantitative Endpoints Reported	Main Mechanistic Evidence	Semi-Quantitative Effect Strength and Evidence Level	Major Evidence Limitations
Flavonoids	Quercetin; luteolin; kaempferol; baicalin/baicalein; myricetin; apigenin	Mainly DSS- and TNBS-induced colitis models; epithelial and macrophage cell models	Body weight loss; DAI; colon length; histological score; MPO activity; TNF-alpha; IL-1beta; IL-6; ROS/MDA; SOD; ZO-1; Occludin; Claudins	Anti-inflammatory and antioxidant effects, barrier protection/repair, gut microbiota modulation, and restoration of SCFAs, bile acids, or tryptophan-related metabolism have been reported for representative flavonoids [27,31,98,99,100,101,102].	Moderate to relatively high	Most evidence is from acute experimental models; direct molecular targets are often unclear; pharmacokinetic and long-term safety evidence remains limited
Polyphenols	Curcumin; resveratrol; chlorogenic acid; EGCG; tannic acid; gallic acid	DSS-induced acute or chronic colitis; TNBS models; intestinal epithelial and immune-cell models	DAI; colon length; histopathological injury; cytokine levels; ROS/MDA; antioxidant enzyme activity; Nrf2/HO-1 expression; intestinal permeability; tight-junction proteins	Anti-inflammatory and antioxidant effects, intestinal barrier protection, gut microbiota modulation, and regulation of microbial metabolites have been reported for representative polyphenols [103,104,105,106,107,108].	Moderate	Effects are often dose- and formulation-dependent; poor bioavailability is a major issue for several candidates; direct causal validation remains insufficient in many studies
Alkaloids	Berberine; palmatine; coptisine	DSS- and TNBS-induced colitis models; intestinal epithelial cell and macrophage models	DAI; colon length; histological score; TNF-alpha; IL-1beta; IL-6; tight-junction proteins; SCFAs; bile acid-related metabolites; microbiota diversity	Anti-inflammatory activity, antioxidant regulation, epithelial barrier protection/repair, and gut microbiota modulation have been reported for representative alkaloids [109,110,111].	Moderate to relatively high, especially for berberine	Mechanistic conclusions are sometimes based on pathway association; clinical evidence and standardized dosing remain limited
Terpenoids and saponins	Ginsenoside Rg1; ginsenoside Rg2; ginsenoside Rg3; glycyrrhizic acid-related complexes; ursolic acid; oleanolic acid derivatives	Mainly DSS-induced colitis models; some immune-cell or epithelial-cell validation models	DAI; body weight; colon length; histological score; TNF-alpha; IL-1beta; IL-17; macrophage activation markers; apoptosis-related proteins; microbiota and tryptophan metabolites	Barrier protection/repair, gut microbiota modulation, and restoration of SCFAs, bile acids, or tryptophan-related metabolism have been reported for representative terpenoids and saponins [34,112,113,114,115].	Moderate	Evidence strength differs markedly among individual compounds; fewer studies include target-binding assays, genetic rescue, or long-term relapse/fibrosis endpoints
Polysaccharides	Astragalus polysaccharides; Ganoderma lucidum polysaccharide]; Lycium barbarum polysaccharide; citrus-derived polysaccharides/pectin	DSS-induced acute or chronic colitis; AOM/DSS-associated colorectal injury models; microbiota-related intervention models	DAI; colon length; histological score; goblet cell number; MUC2; ZO-1/Occludin; SCFA levels; gut microbiota diversity; Treg/Th17 ratio; inflammatory cytokines	Anti-inflammatory activity, epithelial and mucus-barrier protection/repair, gut microbiota modulation, and restoration of SCFAs, bile acids, or tryptophan-related metabolism have been reported for representative polysaccharides [66,116,117,118].	Moderate	Structural heterogeneity is high; active motifs are often unclear; batch-to-batch reproducibility and quality control are major translational barriers
Plant extracts and traditional herbal formulas	Astragalus extract; Polygonum hydropiper extract; licorice extract; ginger extract; Huangqin decoction; modified Gegen Qinlian decoction	DSS-, TNBS-, and chronic colitis models; AOM/DSS models in some studies	DAI; colon length; histological score; inflammatory cytokines; oxidative stress markers; GPX4/ACSL4; KEAP1/Nrf2; MUC2; tight-junction proteins; microbiota/metabolite profiles	Anti-inflammatory activity, antioxidant regulation, and intestinal barrier protection/repair have been reported for representative plant extracts and herbal preparations [119,120,121,122].	Low to moderate	Active constituents are difficult to define; mechanisms are often inferred from network pharmacology or pathway changes; standardization and batch consistency remain insufficient

Note: The level of supporting evidence was assigned according to the depth of validation rather than efficacy ranking. ‘Low’ evidence refers mainly to phenotypic improvement without clear mechanistic validation; ‘moderate’ evidence indicates consistent phenotypic protection accompanied by pathway-associated molecular changes; ‘relatively high’ evidence requires additional support from dose–response studies, target engagement, genetic or pharmacological rescue, microbiota-causality testing, metabolite supplementation, or receptor-level validation. This classification does not represent confirmed clinical efficacy.

#### 3.1.1. Flavonoids

Flavonoids are among the most extensively investigated natural products in experimental colitis, with representative compounds including quercetin, luteolin, kaempferol derivatives, and baicalin/baicalein. Their protective effects are mainly reflected in suppression of inflammatory signaling, attenuation of oxidative injury, and improvement of epithelial barrier integrity. For example, quercetin has been reported to alleviate DSS-induced colitis by reducing oxidative stress and mucosal injury [98], whereas luteolin and baicalein are more frequently associated with inhibition of NF-κB/TLR4-related inflammatory signaling and restoration of barrier function [123,124]. Overall, flavonoids represent a relatively mature class of natural products in colitis research, although more direct target-validation evidence is still needed to distinguish primary mechanisms from downstream pathway changes.

#### 3.1.2. Polyphenols

Polyphenols, including curcumin, resveratrol, chlorogenic acid, EGCG, and related compounds, have also been widely studied in colitis models. Compared with flavonoids, polyphenols are often discussed in relation to redox regulation, mucosal healing, and microbiota-associated metabolic changes. Curcumin has been reported to improve DSS-induced colitis phenotypes and support barrier recovery, whereas resveratrol and chlorogenic acid are frequently linked to suppression of inflammatory responses [125], regulation of oxidative stress, and activation of antioxidant pathways such as Nrf2/HO-1 [126,127]. The translational interpretation of this class should consider dose, formulation, intestinal exposure, and bioavailability, which are particularly important for curcumin and resveratrol.

#### 3.1.3. Alkaloids

Representative alkaloids in colitis research include berberine, matrine, palmatine, and coptisine. Among them, berberine is one of the most frequently investigated candidates and has been associated with inhibition of inflammatory signaling, restoration of epithelial barrier integrity, and regulation of gut microbiota and bile acid metabolism [128,129]. Matrine and related alkaloids have also been reported to alleviate experimental colitis by suppressing NF-κB/MAPK-related pathways and reducing inflammatory mediator production. Compared with many other small molecules, alkaloids show relatively broad effects on the inflammation–barrier–microbiota axis, but standardized dosing and stronger pharmacokinetic evidence remain important for further development.

#### 3.1.4. Terpenoids and Saponins

Terpenoids and saponins include ginsenosides, glycyrrhizic acid-related compounds, ursolic acid, oleanolic acid, and their derivatives. This class is characterized by relatively diverse biological activities, including immune regulation, epithelial protection, modulation of apoptosis, and regulation of microbiota-related metabolism. For instance, ginsenoside Rg1 has been reported to alleviate DSS-induced colitis while influencing gut microbiota composition and tryptophan metabolism [130], whereas oleanolic acid derivatives have been linked to suppression of inflammatory cytokines and regulation of epithelial cell survival [131]. The evidence for this category is promising but highly compound-specific, and therefore individual compounds should be evaluated separately rather than generalized as a uniform class.

#### 3.1.5. Polysaccharides

Polysaccharides derived from plants, fungi, algae, and other natural sources represent a mechanistically distinct category because their effects are more closely related to gut microbiota remodeling, microbial metabolite production, mucus barrier protection, and immune homeostasis than to direct inhibition of a single molecular target. Representative polysaccharides, such as Astragalus polysaccharides and citrus-derived polysaccharides, have been reported to improve experimental colitis by increasing SCFA-producing bacteria, enhancing SCFA production, restoring tight-junction and mucus-barrier markers, and regulating Treg/Th17 balance [116,132]. Therefore, polysaccharides may be particularly suitable for studies focusing on microbiota–metabolite–host interactions and barrier repair. Their structural heterogeneity and quality-control requirements should be considered when evaluating translational potential.

#### 3.1.6. Plant Extracts and Traditional Herbal Formulas

Plant extracts and traditional herbal formulas constitute an important component of natural product research in colitis because they may exert multi-component and multitarget regulatory effects. Compared with purified compounds, these preparations are more often associated with simultaneous improvement of inflammatory signaling, oxidative injury, epithelial barrier disruption, and microbiota dysbiosis. For example, some plant extracts have been reported to regulate ferroptosis-related pathways, Nrf2-mediated antioxidant responses, and tight-junction protein expression in experimental colitis [120,133]. However, because their active constituents and quality markers are often difficult to define, this category requires stronger chemical standardization and component-function clarification before mechanism-based development can be advanced.

### 3.2. Comparative Evaluation and Prioritization of Natural Product Classes

Although different classes of natural products converge on several common protective effects, including anti-inflammatory activity, antioxidant regulation, epithelial barrier repair, and gut microbiota modulation, their current evidence strength and translational potential are not equivalent. Therefore, they should not be discussed only as parallel categories, but should be compared according to mechanistic maturity, reproducibility of phenotypic protection, feasibility of standardization, and translational readiness.

Among the currently investigated candidates, flavonoids and polyphenols represent the most extensively studied classes. Their major advantage lies in relatively consistent anti-inflammatory and antioxidant effects across experimental colitis models, especially through regulation of NF-κB, MAPK, NLRP3, Nrf2/HO-1, and epithelial barrier-related pathways [134,135]. Representative compounds such as quercetin, luteolin, curcumin, resveratrol, and chlorogenic acid have been repeatedly examined in DSS- or TNBS-induced colitis models [136,137]. However, their translational priority should be considered with caution because many studies remain pathway-associated rather than target-validated, and several polyphenols, especially curcumin and resveratrol, are limited by poor bioavailability and formulation-dependent efficacy [138,139].

Alkaloids, particularly berberine-related compounds, appear to have relatively strong translational potential among small molecules because their effects are repeatedly linked to inflammatory signaling, epithelial barrier restoration, and microbiota–metabolite regulation [140]. Compared with many flavonoids and polyphenols, berberine has been more frequently discussed in relation to gut microbiota, bile acid metabolism, and host metabolic regulation [141]. Nevertheless, the mechanisms of alkaloids are still often inferred from pathway changes, and standardized dosing, pharmacokinetic behavior, and clinical validation remain insufficient [142].

Polysaccharides should be prioritized mainly for microbiota–metabolite and barrier-oriented intervention rather than as classical direct-target small molecules [116]. Their strength lies in their prebiotic-like effects, ability to promote short-chain fatty acid production, regulation of Treg/Th17 balance, and protection of the mucus and epithelial barrier [143]. Therefore, polysaccharides may be particularly promising for remission maintenance or microecological restoration. However, their structural heterogeneity, unclear active motifs, batch-to-batch variability, and difficulty in defining direct molecular targets substantially limit their standardization and drug-development potential [144].

Terpenoids and saponins occupy an intermediate position. Some candidates, especially ginsenosides and oleanolic acid derivatives, show promising effects on inflammatory signaling, immune regulation, apoptosis, and microbiota-related metabolism [80]. However, the evidence is highly compound-specific, and fewer studies have provided rigorous causal validation, long-term outcome assessment, or clinical evidence [131]. Therefore, these compounds can be regarded as promising but still under-prioritized candidates that require deeper mechanistic and translational evaluation [114].

Plant extracts and traditional herbal formulas have the broadest multitarget potential but the weakest standardization basis [49]. Their main advantage is the possibility of multi-component synergy, which may be useful for complex chronic inflammatory diseases such as colitis. However, compared with purified compounds, they face greater challenges in identifying active constituents, controlling batch consistency, defining dose–response relationships, and proving direct mechanisms [120]. Therefore, they may be more suitable at the current stage for adjunctive or formula-based research rather than direct development as single-agent therapeutic candidates.

Overall, based on the current balance between evidence strength and translational feasibility, small molecules with relatively consistent phenotypic efficacy and clearer mechanistic signals, such as berberine, quercetin, luteolin, curcumin, and selected ginsenosides, may deserve higher priority for further target validation and formulation optimization. Polysaccharides should be prioritized for microbiota–metabolite-centered studies, whereas plant extracts and traditional formulas require stronger chemical standardization before their therapeutic potential can be reliably compared.

### 3.3. Critical Evaluation of the Current Evidence

#### 3.3.1. Strengths and Mechanistic Limitations of Current Evidence

Although a large number of natural products have shown protective effects in experimental colitis, the overall strength of the current evidence remains uneven [134]. One major strength of this field is that many compounds act on multiple pathological processes simultaneously, including inflammatory signaling, oxidative stress, epithelial barrier injury, immune-cell dysfunction, and gut microbiota dysbiosis [145]. This multitarget property is particularly attractive for colitis, because the disease is driven by interacting inflammatory, epithelial, microbial, and metabolic abnormalities rather than by a single pathway. In addition, several representative compounds, such as quercetin, curcumin, berberine, ginsenosides, and polysaccharides, have been repeatedly investigated in DSS- or TNBS-induced colitis models, providing relatively consistent phenotypic evidence for their protective effects [146].

A central weakness of the current literature is that phenotypic improvement, pathway association, and direct pharmacological mechanisms are often discussed at the same level. Changes in body weight, colon length, DAI, histological score, cytokine expression, ROS production, or tight-junction proteins demonstrate disease improvement but do not identify the primary mechanism. Similarly, Western blotting, qPCR, immunostaining, molecular docking, and network pharmacology should be regarded as pathway-associated or hypothesis-generating evidence rather than proof of direct target engagement. Stronger mechanistic claims require direct binding assays, target engagement in cells or tissues, genetic loss- and gain-of-function experiments, site-directed mutagenesis, inhibitor rescue, or phenotype rescue experiments.

Therefore, a microbiota-dependent mechanism should not be claimed unless compositional changes are linked to functional outcomes through at least one causal validation strategy, such as antibiotic depletion followed by phenotype loss, FMT-mediated phenotype transfer, defined-strain supplementation, germ-free or gnotobiotic validation, metabolite rescue, or receptor/pathway blockade.

#### 3.3.2. Inconsistent and Inconclusive Evidence

Some findings across the literature are not fully consistent [147]. Different studies often use different colitis models, animal strains, sex, doses, routes of administration, intervention windows, and endpoint definitions [148]. As a result, the same class of natural products may show strong anti-inflammatory effects in acute DSS-induced epithelial injury but much weaker or untested effects in chronic relapsing colitis, fibrosis, or colitis-associated tumorigenesis [149]. In addition, some compounds are repeatedly described as anti-inflammatory, antioxidant, barrier-protective, and microbiota-modulating agents, but the relative contribution of each mechanism is rarely compared within the same experimental design [150].

Clinical studies provide several examples showing that the evidence for natural products in colitis is not uniformly positive. For curcumin, although some studies support its adjunctive role in maintaining remission in UC, low-dose oral curcumin at 450 mg/day failed to induce remission in mild-to-moderate UC [151,152]. Moreover, a recent meta-analysis showed that curcumin improved clinical remission, but its effect on endoscopic remission was not statistically significant and substantial heterogeneity was present [153]. These findings suggest that the efficacy of curcumin may depend on dose, formulation, disease stage, and endpoint selection.

Similar inconsistency has been observed for herbal medicines. A systematic review and meta-analysis of randomized controlled trials in active UC found that Indigo naturalis significantly improved clinical response, whereas Curcuma longa did not significantly improve clinical response and Andrographis paniculata did not significantly improve either clinical response or clinical remission [154]. Aloe vera gel also showed preliminary positive effects in a small short-term randomized trial, but the evidence remains insufficient for firm conclusions because larger and replicated trials are lacking [155]. In addition, Boswellia serrata has been reported to improve symptoms in small UC studies, but its role in relapse prevention remains unconvincing. These examples indicate that natural products should not be presented as uniformly effective; rather, their therapeutic value appears to be compound-specific, dose-dependent, endpoint-dependent, and strongly influenced by study design [156].

#### 3.3.3. Translational Uncertainty: Pharmacokinetics, Safety, and Standardization

Finally, the translational value of current evidence remains limited by insufficient pharmacokinetic, safety, and standardization data [157]. Many natural products show activity in animal models at relatively high doses, but their intestinal exposure, systemic absorption, metabolic stability, active metabolites, and long-term safety remain unclear [158]. This problem is particularly important for plant extracts and traditional herbal formulas, in which the active constituents, batch-to-batch reproducibility, and quality-control standards are often insufficiently defined. Therefore, future studies should move beyond descriptive efficacy testing and adopt a more rigorous framework that integrates dose–response assessment, direct target validation, microbiota-causality testing, long-term safety evaluation, and standardized product characterization. From a regulatory and product-development perspective, natural products, especially plant extracts and traditional herbal formulas, require raw material authentication, standardized extraction procedures, chemical fingerprints, active or analytical markers, impurity profiling, batch-to-batch consistency assessment, and predefined quality-control criteria. Without these elements, positive pharmacological findings are difficult to translate into reproducible and clinically acceptable products [159].

## 4. Key Limitations in Current Research

### 4.1. Overreliance on a Single Animal Model

Although the number of preclinical studies has increased rapidly, the current evidence for natural products in the treatment of colitis still relies heavily on experimental colitis models, particularly the DSS-induced model [160,161]. The DSS model is widely used because of its simple operation, good reproducibility, and obvious phenotypic features; however, it mainly reflects acute chemical epithelial injury and secondary inflammatory responses and therefore cannot fully recapitulate the complex chronic relapsing course, immune heterogeneity, and long-term tissue remodeling observed in clinical colitis [162,163]. Consequently, positive findings obtained solely from the DSS model cannot be directly extrapolated to imply efficacy across all pathological stages of ulcerative colitis.

In addition, many studies are conducted using only a single model, a single sex of animals, or even a single observation window, without complementary validation in TNBS, oxazolone, AOM/DSS, genetically susceptible models, or chronic recurrent models. As a result, although some natural products perform well in short-term control of inflammation, their true effects on chronic inflammation, fibrosis tendency, recurrent disease course, and carcinogenic risk remain unclear. Therefore, future studies should gradually move from “single acute model validation” toward an evaluation framework based on “multiple models, multiple stages, and multiple endpoints.”

### 4.2. Unclear Active Constituents and Direct Molecular Targets

Although many studies have demonstrated that certain natural products can improve colitis phenotypes, work that clearly identifies which component plays the key role, what the direct molecular target is, and whether molecular binding is actually established remains limited [164,165]. This issue is particularly prominent for plant extracts and traditional herbal formulas. Many studies remain at the level of showing that an extract alleviates inflammation and tissue injury, but they do not sufficiently explain the core bioactive constituents, the synergistic relationships among components, or the selectivity of target engagement.

Even in studies on single compounds, it is common to see targets predicted by network pharmacology or molecular docking without genuine binding validation or functional rescue experiments [166,167,168,169]. In other words, many papers can show that a natural product is associated with changes in a certain pathway, but they cannot further prove that the pathway change is the direct pharmacological basis of its action. From the perspective of drug development, unclear active constituents and direct targets will substantially limit subsequent structural optimization, formulation development, and extension to broader indications. For single compounds, a minimal target-validation pipeline should include molecular docking or network pharmacology only as hypothesis-generating tools; pull-down assays, CETSA, SPR, MST, DARTS, or thermal proteome profiling for direct binding or target engagement; siRNA/CRISPR-mediated knockdown, overexpression, or pharmacological inhibition to test functional relevance; and mutant rescue or pathway rescue experiments to confirm causality. For extracts and herbal formulas, active fractions and chemical markers should first be defined before target-level conclusions are made.

### 4.3. Insufficient Evaluation of Bioavailability, Pharmacokinetics, and Safety

Natural products show broad potential in preclinical studies, but once they enter translational development, the first challenge is often not “whether they are effective,” but “whether they are usable.” Many bioactive compounds suffer from poor solubility, inadequate stability, limited oral absorption, rapid metabolism, and insufficient local exposure in the intestine. These factors directly restrict the effective in vivo concentration and duration of action. Classic natural products such as curcumin and resveratrol are typical examples: although their effects in vitro and in animal experiments are relatively remarkable, bioavailability problems have long hindered their further application.

At the same time, natural products are often assumed to be “highly safe,” but this impression is not equivalent to systematic toxicological evidence. Many studies report only short-term protective effects and lack assessments of dose windows, long-term exposure, drug interactions, and chronic safety. For plant extracts and traditional herbal formulas, batch-to-batch variation and fluctuations in composition may further magnify this problem. Therefore, inadequate pharmacokinetic and safety studies remain a major bottleneck preventing natural products from moving from the laboratory to the clinic. Future studies should therefore report not only administered dose but also intestinal exposure, systemic absorption, metabolic stability, active metabolites, tissue distribution, and pharmacokinetic/pharmacodynamic relationships. For poorly absorbed compounds, local colonic exposure may be more relevant than plasma concentration and should be measured directly where possible.

### 4.4. Insufficient Attention to Long-Term Outcomes: Relapse, Fibrosis, and Safety

Another important limitation of current research is that most studies focus on short-term anti-inflammatory effects, whereas long-term outcomes that are more relevant to clinical colitis remain insufficiently evaluated [147]. IBD is a chronic and relapsing disease, and successful intervention should not only reduce acute inflammatory injury but also help maintain remission, prevent relapse, limit chronic tissue remodeling, and avoid long-term adverse effects [170]. However, many studies of natural products still rely on acute DSS-induced colitis models with short observation periods, usually assessing body weight, disease activity index, colon length, histological injury, and inflammatory cytokines within a limited treatment window. Although these endpoints are useful for evaluating acute protection, they cannot fully determine whether a candidate can prevent relapse or sustain mucosal healing after treatment withdrawal.

Fibrosis and chronic tissue remodeling are also rarely assessed. Long-standing intestinal inflammation can lead to extracellular matrix deposition, fibroblast activation, collagen accumulation, and intestinal stricture formation [171]. However, most natural product studies evaluate only acute histological injury and inflammatory infiltration, without examining fibrotic markers such as collagen deposition, α-SMA, fibronectin, TGF-β/Smad signaling, or matrix metalloproteinase activity. Therefore, it remains uncertain whether natural products can truly modify chronic disease progression or only transiently suppress acute inflammation.

Long-term safety is another key translational issue. Natural products are often assumed to be safe because they are naturally derived, but this assumption is not equivalent to systematic toxicological evidence [172]. High-dose or long-term administration may cause unexpected hepatic, renal, metabolic, or immune-related effects, and plant extracts or traditional herbal formulas may also vary in composition across batches. Therefore, future studies should include repeated-dose toxicity assessment, hematological and serum biochemical analysis, histological examination of major organs, evaluation of drug–drug interactions, and safety monitoring after prolonged exposure. Only when relapse prevention, fibrosis-related outcomes, and long-term safety are evaluated together can the translational value of natural products for colitis be more accurately judged.

### 4.5. Weak Clinical Evidence and Insufficient Standardization

Compared with preclinical research, high-quality clinical evidence for natural products in colitis remains clearly insufficient. Although a small number of natural medicines or herbal preparations have entered clinical trials, and some systematic reviews and meta-analyses have suggested the adjunctive therapeutic potential of certain herbal medicines in active ulcerative colitis, the overall clinical evidence remains weak due to small sample sizes, substantial heterogeneity in study design, and lack of uniformity in intervention protocols and outcome measures.

In addition, research on natural products still faces inadequate standardization, including inconsistency in raw material sources, differences in extraction procedures, unstable levels of active constituents, and non-unified quality-control criteria. These problems reduce comparability among studies and make it difficult to establish clear evidence hierarchies and recommendation systems. Therefore, to promote the clinical application of natural products in colitis, it is necessary to simultaneously strengthen the standardization of study design and the standardization of product quality. Clinical evidence should also be reported in an endpoint-specific manner. Clinical response, clinical remission, endoscopic remission, steroid-free remission, relapse prevention, and mucosal healing represent different levels of therapeutic benefit and should not be treated as interchangeable outcomes.

## 5. Future Directions and Strategic Recommendations

### 5.1. Strengthening Veterinary and Comparative Relevance

Future studies should more explicitly evaluate natural products in veterinary species and clinically relevant animal intestinal disorders. For companion animals, studies should focus on canine and feline chronic enteropathies, including clinical activity scores, fecal consistency, endoscopic and histological changes, microbiota composition, metabolite profiles, and response to diet or adjunctive interventions. For horses, studies should consider acute colitis and diarrhea, with attention to fecal water content, systemic inflammation, dehydration, endotoxemia-related markers, hindgut dysbiosis, and recurrence. For food-producing animals, particularly weaned pigs and calves, natural products should be evaluated using endpoints such as diarrhea incidence, growth performance, feed efficiency, intestinal morphology, barrier function, immune status, microbiota–metabolite profiles, antimicrobial-use reduction, and target-animal safety. These veterinary endpoints are essential for determining whether natural products have practical value beyond experimental murine colitis models.

### 5.2. From Phenotypic Improvement to Direct Target Validation

Future research on natural products in colitis should reduce reliance on the common pattern of drawing conclusions from “symptom improvement plus pathway change,” and instead place greater emphasis on the discovery and validation of direct molecular targets. For single compounds, causal strength should be enhanced through binding assays, genetic interventions, and functional rescue experiments. For plant extracts and herbal formulas, efforts should focus on the separation of active components, optimization of key component combinations, and clarification of their chemical basis. Only when targets are clearly defined can subsequent optimization and clinical translation become truly actionable.

### 5.3. From Preclinical Research to Standardized and Translatable Development

The next stage of natural product development should not remain at the level of “discovering more effective compounds,” but should shift toward a standardized, reproducible, and translatable development model. This includes establishing unified extraction and quality-control standards, improving pharmacokinetic and safety evaluation systems, optimizing administration routes and delivery strategies, and promoting high-quality randomized controlled clinical trials. Only when a complete chain is formed among chemical basis, pharmacological mechanisms, formulation performance, and clinical evidence can natural products truly evolve from “promising candidates” in colitis research into clinically usable intervention resources.

### 5.4. Translational Positioning of Natural Products as Adjunctive Strategies

Although natural products still face many challenges when considered as standalone drug development candidates, their potential as combination therapies or adjunctive treatment strategies has attracted increasing attention. Because natural products usually possess multitarget regulatory properties, and some of their constituents complement existing drugs in suppressing inflammation, supporting barrier repair, and improving microbial homeostasis, combining them with 5-aminosalicylic acid, corticosteroids, biologics, or small-molecule drugs may theoretically achieve dose reduction with maintained efficacy, fewer adverse effects, and prolonged remission.

More importantly, combination therapy may help circumvent the practical limitation that some natural products are insufficiently effective on their own but may function well as synergistic regulators. Therefore, a key future direction is not to simply position natural products as “alternative therapies” in opposition to existing drugs, but rather to explore their rational roles in integrated disease management, for example as adjuncts for remission maintenance, tools for supporting microbiota homeostasis, or promoters of mucosal healing.

### 5.5. Delivery-System Optimization and Colon-Targeted Formulation Development

To overcome the problems of low bioavailability, poor stability, and insufficient local enrichment of natural products, delivery strategies based on nanocarriers, micro-/nanomicelles, hydrogels, liposomes, and colon-targeted release systems have increasingly become research hotspots in recent years. Relevant reviews have pointed out that these novel delivery systems can not only improve the stability and absorption efficiency of natural products in the gastrointestinal environment but also enhance their targeted accumulation at inflammatory sites, thereby improving efficacy and reducing systemic adverse effects.

From a translational perspective, the significance of novel delivery systems lies not merely in “delivering the drug,” but in facilitating the transition of natural products from “preclinical bioactive compounds” to “druggable candidate formulations.” Future directions worth particular attention include colon-specific release systems, responsive carriers sensitive to the intestinal microenvironment, combined delivery systems with probiotics or postbiotics, and intelligent carrier designs capable of simultaneously achieving local retention and mucosal penetration.

## Data Availability

No new data were created or analyzed in this study. Data sharing is not applicable to this article.
